# Social Participation, Social Support, and Social Policy Among Older Adults

**DOI:** 10.1093/geroni/igaa057.3531

**Published:** 2020-12-16

**Authors:** Linda Waite

## Abstract

The social world is closely linked to other dimensions of health, including physical health and illness, physical functioning, cognition and emotional well-being, and these links may change across generations and may depend on social and policy context. The papers in this symposium focus on these links. Carr examines the associations between productive engagement in later life and perceptions of social support and interactions with friends and family. She finds that volunteer engagement is associated with greater perceived social support and interaction with friends and family but not with support from spouse. Waite, Duvoisin and Kotwal measure changes in social participation between the Silent Generation cohort, born between1938 and1947, and the Baby Boom cohort, born from1948-1958. They find find that the gender differences shown in the Silent Generation cohort are reduced among those born during the Baby Boom. Azar examines the moderating role of social policy, particularly defamilization, on the link between loneliness and health, using data from30 European countries and the U.S. Choi compares marital and partnership status, social support and strain in Silent Generation vs. Baby Boom cohorts. Her results suggest that those born during the Baby Boom are embedded in looser social relationships compared to their older counterparts. Together, these papers point to the importance of considering various dimensions of social life, gender, and context, including historical time and the life cycle, in understanding how the social world acts to affect well-being.

